# Chest pain mimicking pulmonary embolism may be a common presentation of COVID‐19 in ambulant patients without other typical features of infection

**DOI:** 10.1111/joim.13267

**Published:** 2021-03-13

**Authors:** S. R. Harrison, J. R. L. Klassen, C. Bridgewood, A. Scarsbrook, H. Marzo‐Ortega, D. McGonagle

**Affiliations:** ^1^ From the Leeds Institute of Rheumatic and Musculoskeletal Medicine (LIRMM) University of Leeds Leeds UK; ^2^ Department of Radiology Leeds Teaching Hospitals Leeds UK; ^3^ Leeds Institute of Medical Research University of Leeds Leeds UK; ^4^ National Institute for Health Research (NIHR) Leeds Biomedical Research Centre (BRC) Leeds Teaching Hospitals Leeds UK

**Keywords:** noncardiac chest pain, thromboembolism, radiology, infectious disease

## Abstract

**Background:**

Radiological and pathological studies in severe COVID‐19 pneumonia (SARS‐CoV‐2) have demonstrated extensive pulmonary immunovascular thrombosis and infarction. This study investigated whether these focal changes may present with chest pain mimicking pulmonary emoblism (PE) in ambulant patients.

**Methods:**

CTPAs from outpatients presenting with chest pain to Leeds Teaching Hospital NHS Trust 1st March to 31 May 2020 (*n* = 146) and 2019 (*n* = 85) were compared. Regions of focal ground glass opacity (GGO), consolidation and/or atelectasis (parenchymal changes) were determined, and all scans were scored using British Society for Thoracic Imaging (BSTI) criteria for COVID‐19, and the 2020 cohort was offered SARS‐CoV‐2 antibody testing.

**Results:**

Baseline demographic and clinical data were similar between groups with absence of fever, normal lymphocytes and marginally elevated CRP and D‐Dimer values. Evidence of COVID‐19 or parenchymal changes was observed in 32.9% (48/146) of cases in 2020 compared to 16.5% (14/85) in 2019 (*P* = 0.007). 11/146 (7.5%) patients met BSTI criteria for COVID‐19 in 2020 compared with 0/14 in 2019 (*P* = 0.008). 3/39 patients tested had detectable COVID‐19 antibodies (2 with parenchymal changes and 1 with normal parenchyma) however 0/6 patients whose CTPA met BSTI criteria “likely/suspicious for COVID‐19” and attended antibody testing were SARS‐CoV‐2 antibody positive.

**Conclusions:**

32.8% ambulatory patients with suspected PE in 2020 had parenchymal changes with 7.5% diagnosed as COVID‐19 infection by imaging criteria, despite the absence of other COVID‐19 symptoms. These findings suggest that localized COVID‐19 pneumonitis with immunothrombosis occurs distal to the bronchiolar arteriolar circulation, causing pleural irritation and chest pain without viraemia, accounting for the lack of fever and systemic symptoms.

## Graphical abstract

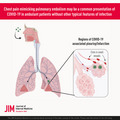

## Introduction

In December 2019, the first cases of a novel human coronavirus (COVID‐19/ SARS‐CoV‐2) emerged in the Wuhan province of China. Since then, COVID‐19 infection spread globally, with over 33 000 000 infections and over 1 000 000 deaths to date [1]. Typically, the most severe cases of COVID‐19 present with a diffuse pneumonia characterized radiologically by bilateral ground glass opacities (GGOs) with predilection for the lung periphery and associated areas of consolidation and atelectasis [2]. Such cases are associated with a burden of thrombosis hitherto unheard of in the context of other viral pneumoniae, with immunovascular thrombosis/pulmonary emboli (PE) seen in approximately one‐third of hospitalized patients, and a still greater incidence in those admitted to intensive care [3, 4, 5]. Postmortem studies of patients succumbing to severe COVID‐19 pneumonia have shown very extensive in situ thrombosis of the pulmonary capillary networks and adjacent pulmonary vasculature with associated extensive pulmonary infarction [6]. Also, a high frequency of PE or in situ thrombosis has been demonstrated in severe COVID‐19 pneumonia cases as assessed by CT pulmonary angiogram (CTPA) [6, 7, 8].

COVID‐19 pneumonia can be completely asymptomatic or may present with severe shortness of breath, hypoxaemia and bronchopneumonia [2]. Other viral pneumonias, including those caused by other coronavirus infections, such as severe acute respiratory syndrome and Middle‐Eastern respiratory virus sometimes present with pleuritic chest pain [9]. A recent systematic review of the diagnosis and management of COVID‐19 did not recognize chest pain as a symptom of COVID‐19 [2]. However, much of the existing research on COVID‐19 focuses on more severe cases of disease, and less is known about the clinical manifestations of milder disease presentations, which may not present with the classical triad of dry cough, fever and loss of taste. Also, diagnostic testing including throat swabbing and antibody testing are less likely to be positive in subjects with milder disease [10, 11].

Given the strong link with immunothrombosis and the peripheral distribution of lung parenchymal changes associated with COVID‐19, we hypothesized that a proportion of mild COVID‐19 cases may present with chest pain secondary to localized pulmonary infarction and subsequent irritation of the adjacent lung pleura. Due to the focal nature of the infection, these patients would not necessarily present with classical COVID‐19 symptoms of breathlessness, dry cough, fever and loss of taste; instead, they may present to the emergency department with pleuritic‐type chest pain. Our aims were therefore to investigate outpatients presenting with suspected PE symptoms, but where PE was not confirmed by CTPA during a three month period in 2020 at the peak of the first wave of the pandemic in the UK and also over the corresponding timeframe in 2019. The clinical and radiological features of these cases and later SARS‐CoV‐2 antibody serology in those who were tested are reviewed.

## Materials and methods

This was an approved retrospective audit of service delivery at our institution, and formal ethical approval was not required. Electronic records of outpatients attending Leeds Teaching Hospitals (LTH) NHS Trust from 1 March to 31 May 2020 (corresponding to the first peak of the COVID‐19 pandemic in our region) were reviewed. Records of patients presenting over the same time period in 2019 (prepandemic) were used as a comparator. Inclusion criteria were clinically stable patients presenting with chest pain who underwent a CTPA on the basis that PE was the likely primary diagnosis. Exclusion criteria were haemodynamically unstable patients, patient requiring supplementary oxygen, those requiring admission due to the severity of their illness and those with a known diagnosis of COVID‐19.

Patients were first divided into PE‐positive and PE‐negative with the latter further categorized based on whether or not there were associated areas of pulmonary parenchymal change (including either GGOs, atelectasis and/or consolidation). CTPAs were reviewed by two observers, including an experienced Consultant Radiologist. In addition, 30 random CTPA images and their corresponding reports were reviewed with no discrepancies found with original reports.

The classification system used for reports is outlined below:

1. “Likely/suspicious for COVID‐19" pneumonia as per the British Society for Thoracic Imaging (BSTI) reporting criteria used by UK radiologists during the COVID‐19 pandemic [12].
2. Parenchymal changes.
3. Alternative pathology (including heart failure, pleural effusion, or malignancy, etc.)
4. No explanation for symptoms with completely normal thoracic imaging.

Relevant demographic and clinical data were collected for both cohorts including age, sex, smoking status, presenting symptoms, basic clinical observations, haemoglobin (Hb), white blood cell count (WBC), neutrophil count, lymphocyte count, D‐Dimer, high‐sensitive troponin‐I and C‐Reactive Protein (CRP) from clinical records, in addition to Wells PE scores which were calculated retrospectively based on the documented clinical findings when missing from the clinical notes. Chest radiographs (CXRs) were performed in the majority of patients from both 2019 and 2020 (206/232) prior to CTPA as per our hospital protocols.

COVID‐19 PCR testing was not performed routinely on the 2020 cohort as Institutional policy at the time of the audit mandated that nasopharyngeal swabs were only performed on inpatients with suspected COVID‐19. COVID‐19 antibody testing (Siemens total IgM/IgG assay to the SARS‐CoV‐2 spike glycoprotein) was retrospectively requested in the cases from 2020 that had positive CTPA scans in agreement with local hospital governance and subsequent national policy [13].

Statistical analysis was performed using SPSS v26. Mean/standard deviation was used for normally distributed variables and median/interquartile range (IQR) for skewed data. Significance was determined using chi‐squared tests for categorical variables.

## Results

### Study population and baseline clinical data

**Fig. 1:**
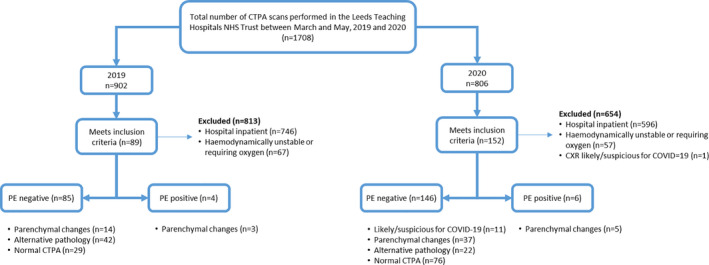
Flowchart illustrating patient selection for the study. Key: CTPA = CT pulmonary angiogram; PE = pulmonary embolus.

Overall, our institution performed 806 CTPAs between 1 March and 31 May 2020 and 902 over the same time period in 2019 (Fig. 1). This included 146 outpatients in 2020 and 85 in 2019 (Fig. 1). More outpatient CTPA scans were performed in 2020, with outpatient scans accounting for 18.1% of all CTPAs performed in our trust in 2020 compared with just 9.4% in 2019 (*P* < 0.001).

The basic demographic data for these patients did not significantly differ between 2019 and 2020 cohorts (Table 1, all *P* > 0.05). In 2020, the average age was 52.8 years [±18.3 years (one standard deviation)] and almost two‐thirds of our patients were female (64.4%, *n* = 94/146), compared with just 54.1% (*n* = 46/85) in 2019, although this was not statistically significant (*P* = 0.126). 28.6% (*n* = 20/70) were current smokers, and 60.3% (*n* = 85/141) of patients had one or more comorbidities including cardiovascular disease (e.g. ischaemic heart disease or hypertension), respiratory disease (e.g. chronic obstructive pulmonary disease), immunosuppression therapy or malignancy.

**Table 1:** Baseline clinical and laboratory test results for all patients

	Overall (*n* = 231)	2019 Cohort (*n* = 85)	2020 Cohort (*n* = 146)	Patients with parenchymal changes (n = 62)
Age (mean ± SD)	53.9 ± 18.2 (*n* = 231)	55.7 ± 17.9 (*n* = 85)	52.8 ± 18.3 (*n* = 146)	55.8 ± 17.9 (*n* = 62)
Sex (% male, no.)	39.4% (*n* = 91/231)	45.9% (*n* = 39/85)	35.6% (*n* = 52/146)	51.6% (*n* = 32/62)
Current smoker (%, no)	23.3% (*n* = 27/116)	15.6% (*n* = 7/45)	28.6% (*n* = 20/70)	19.0% (*n* = 4/21)
Comorbidities associated with increased risk of mortality from COVID‐19*	61.4% (*n* = 137/223)	63.4% (*n* = 52/82)	60.3% (*n* = 85/141)	63.3% (*n* = 38/60)
Chest pain (%, no)	100.0% (*n* = 231/231)	100.0% (*n* = 85/85)	100.0% (*n* = 146/146)	100.0% (*n* = 62/62)
SOB (%, no)	62.8% (*n* = 145/231)	65.9% (*n* = 56/85)	61.0% (*n* = 89/146)	71.0% (*n* = 44/62)
Documented evidence of fever (%, no)	2.7% (*n* = 6/224)	2.5% (*n* = 2/81)	2.7% (*n* = 4/143)	4.8% (*n* = 3/62)
Loss of taste (%, no)	0.9% (*n* = 2/229)	0% (*n* = 0/85)	1.4% (*n* = 2/146)	0.0% (0/62)
Dry cough (%, no)	18.2% (*n* = 42/231)	12.9% (*n* = 11/85)	21.2% (*n* = 31/146)	21.0% (*n* = 13/62)
Productive cough (%, no)	14.7% (*n* = 34/231)	20.0% (*n* = 17/85)	11.6% (*n* = 17/146)	17.7% (*n* = 11/61)
Palpitations (%, no)	6.5% (*n* = 15/231)	5.9% (*n* = 5/85)	6.8% (*n* = 10/146)	3.2% (*n* = 2/62)
Dizziness (%, no)	4.8% (*n* = 11/231)	2.4% (*n* = 2/85)	6.2% (*n* = 9/146)	6.5% (*n* = 4/62)
DVT features (%, no)	9.5% (*n* = 22/231)	7.1% (*n* = 6/85)	11.0% (*n* = 16/146)	12.9% (*n* = 8/62)
CXR showing no acute pathology (%, no)	80.5% (*n* = 165/205)	64.7% (*n* = 55/74)	83.3% (*n* = 110/132)	69.4% (*n* = 43/62)
Wells PE score (median, IQR)	1.75 (0–4.5) (*n* = 232)	3.0 (0.0–4.5) (*n* = 85)	1.0 (0.0–4.0) (*n* = 146)	1.3 (0.0–4.1) (*n* = 62)
HR (mean ± SD) (beats per minute)	88.6 ± 19.2 (*n* = 225)	88.1 ± 20.3 (*n* = 81)	89.0 ± 18.7 (*n* = 144)	89.5 ± 19.5 (*n* = 62)
SBP (mean ± SD) (mmHg)	136.3 ± 23.9 (*n* = 223)	134.2 ± 24.8 (*n* = 81)	137.5 ± 23.3 (*n* = 142)	139.8 ± 24.8 (*n* = 61)
DBP (mean ± SD) (mmHg)	81.8 ± 14.9 (*n* = 223)	81.9 ± 15.0 (*n* = 81)	81.7 ± 14.9 (*n* = 142)	86.1 ± 18.0 (*n* = 61)
Temp (mean ± SD) (^o^C)	36.8 ± 0.6 (*n* = 224)	36.8 ± 0.5 (*n* = 81)	36.8 ± 0.6 (*n* = 143)	36.8 ± 0.63 (*n* = 62)
RR (mean ± SD) (breaths per minute)	19.0 ± 2.9 (*n* = 220)	19.2 ± 3.0 (*n* = 79)	18.9 ± 2.8 (*n* = 141)	19.5 ± 3.3 (*n* = 61)
SpO_2_ (mean ± SD)	97.1 ± 2.2 (*n* = 221)	96.7 ± 2.4 (*n* = 79)	97.4 ± 2.0 (*n* = 142)	97.0 ± 2.0 (*n* = 61)
Hb (mean ± SD) (g L^−1^)	130.3 ± 18.4 (*n* = 230)	130.9 ± 20.0 (*n* = 84)	130.0 ± 17.5 (*n* = 146)	132.1 ± 18.0 (*n* = 62)
WBC (mean ± SD) (10^9^/L)	8.58 ± 3.27 (*n* = 230)	9.3 ± 3.7 (*n* = 84)	8.2 ± 2.9 (*n* = 146)	8.8 ± 3.4 (*n* = 62)
neut (mean ± SD) (10^9^/L)	6.10 ± 3.20 (*n* = 229)	6.9 ± 3.5 (*n* = 84)	5.6 ± 2.9 (*n* = 146)	6.3 ± 3.5 (*n* = 62)
lymph (mean ± SD) (10^9^/L)	1.70 ± 1.05 (*n* = 229)	1.6 ± 1.0 (*n* = 84)	1.8 ± 1.1 (*n* = 146)	1.7 ± 0.80 (*n* = 62)
D‐Dimer positive (%, no)**	51.0% (*n* = 103/202)	47.2% (*n* = 34/72)	53.1% (*n* = 69/130)	55.6% (*n* = 30/54)
D‐Dimer (median, IQR) (ng mL^−1^)	401.0 (272.0–764.0) (*n* = 201)	349.5 (249.0–632.5) (*n* = 72)	409.0 (276.0–826.0) (*n* = 129)	417.0 (270.8–875.0) (*n* = 54)
Trop (median, IQR) (ng mL^−1^)	4.40 (2.5–10.4) (*n* = 151)	5.0 (2.9–21.1) (*n* = 37)	4.2 (2.5–8.3 (*n* = 114)	4.7 (2.5–9.3) (*n* = 47)
CRP (median, IQR) (mg L^−1^)	11.7 (5.0–46.0) (*n* = 167)	27.7 (5.0–74.3) (*n* = 56)	6.7 (5.0–36.0) (*n* = 111)	12.1 (5.0–40.3) (*n* = 48)

Key: CRP, C‐Reactive Protein; CXR, Chest X‐Ray; DBP, diastolic blood pressure; DVT, deep vein thrombosis; Hb, haemoglobin; HR, heart rate; IQR, interquartile range; lymph, lymphocyte count; neut, neutrophil count; RR, respiratory rate; SBP, systolic blood pressure; SD, standard deviation; SOB, shortness of breath; SpO_2_, oxygen saturations; Temp, temperature; Trop, Troponin‐I; WBC, white blood cell count.

* This includes cardiovascular disease, respiratory disease, immunosuppression or malignancy.

** Cut off value for positive D‐Dimer using our assay was >400 ng mL^−1^.

The symptoms and investigations for all patients are reported in Table 1. In 2020, only 2.7% (4/143) had a documented fever, and less than 5% of patients reported viral prodromal symptoms including headache (0/146), myalgia/ arthralgia (7/146), lethargy (6/146), sore throat (4/146) and loss of taste (0/146). Chest pain was universally reported (100.0%, *n* = 146/146), and shortness of breath (61.0%, *n* = 89/146) and/or productive cough (11.6%, *n* = 17/146) were common. A dry cough was reported by 21.2% (*n* = 31/146). Clinical observations, including heart rate, respiratory rate, temperature, blood pressure and oxygen saturations were within normal range. Lymphocyte counts were normal [lymphocytes 1.76 × 10^9^ ± 1.07 (*n* = 145/146)], whilst D‐Dimer and CRP values were slightly elevated 409.0 ng mL**^−1^** (276.0–826.0, *n* = 129/146) and 6.7 mg L^−1^ (5.0–36.0, *n* = 111/146), respectively].

In 2020, 131/146 patients (89.7%) had a baseline CXR, and of these, 15.9% (*n* = 21/132) were abnormal, including lobar pneumonia in 7/22, small effusions in 5/22, other diagnoses in 2/22 and parenchymal changes in 7/22. Findings on CXR from 2019 were similar, with equivalent numbers of patients having abnormal baseline CXRs each year (*P* = 0.155). CXR findings were generally minor with the physicians progressing to CTPA on the basis the CXR did not adequately explain the clinical picture.

### Pulmonary parenchymal findings

**Fig. 2:**
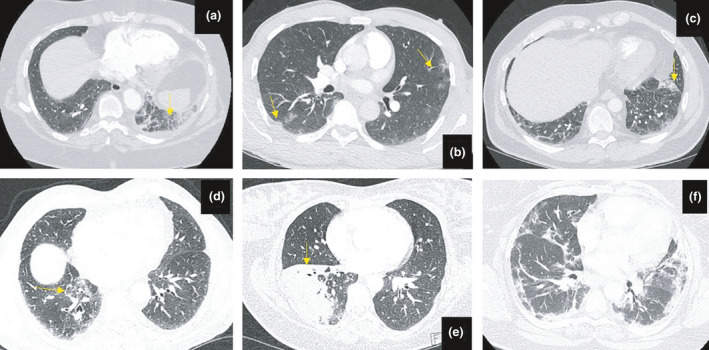
Examples of lung parenchymal changes on CT pulmonary angiogram (CTPA). Parenchymal changes are indicated by the yellow arrows. a = 2020 CTPA positive for PE with associated parenchymal changes [ground glass opacities (GGOs)]; b = 2020 CTPA negative for PE with multiple peripherally distributed GGOs; c = 2020 CTPA negative for PE with a single peripheral GGO; d = 2019 CTPA negative for PE with peri‐bronchial GGOs; e = 2020 CTPA negative for PE with right lower lobe consolidation; f = an example of more extensive parenchymal changes in a patient who had a positive PCR test for COVID‐19.

In the 2020 cohort, 32.9% (*n* = 48/146) of outpatients had lung parenchymal changes on CTPA but no evidence of PE; this included 37 patients with nonspecific parenchymal changes and 11 (7.5%) with features that were “likely/suspicious for COVID‐19 pneumonia” (Fig. 1), whereas in 2019 only 16.5% (*n* = 14/85) exhibited similar parenchymal changes (*P* = 0.007; Table 2). Examples of pulmonary changes are shown in Fig. 2. All the patients with parenchymal changes on their CTPA had similar baseline demographic and clinical characteristics as those without parenchymal changes (Table 1, all *P* > 0.05). None of the 7.5% (11/146) patients whose CTPA was reported as “likely/suspicious for COVID‐19” were suspected of having COVID‐19 prior to CTPA, with 8 having a normal CXR and 3 having minor nonspecific areas of collapse. The BSTI criteria for COVID‐19 pneumonia were retrospectively applied to the 14 CTPAs from 2019 with none classified as “likely/suspicious for COVID‐19”, though 13/14 were classified as “indeterminate for viral infection” by the reviewing radiologist in 2020 (AS) (*P* = 0.008). Removing the 11 likely COVID‐19 cases from 2020 left 25.3% (*n* = 37/146) patients with parenchymal changes and the same 16.5% (*n* = 14/85) cases in 2019 (*P* = 0.061, Table 2).

**Table 2:** Summary of all institutional CTPA scans performed from 1 March to 31 May in 2019 and 2020

	2019 Cohort	2020 Cohort	*P* values
Total CTPAs performed during the study period* *Includes medical unstable inpatients and stable outpatients’ scans	902	806	
Medially stable outpatients undergoing CTPAs to exclude PE	89	154	
CTPA positive, parenchymal changes positive	3	5	
CTPA positive, parenchymal changes negative	1	1	
CTPA negative parenchymal changes positive	14	48	0.007
(i) Radiologically likely COVID	0	11	
(ii) Parenchymal changes	14	37	0.061
CTPA negative parenchymal changes negative	71 [nil acute, 40.8% (*n* = 29)] [other, 59.2%, *n* = 42)]	98 [nil acute, 77.6% (*n* = 76)] [other, 32.4% (*n* = 22)]	

Key: CTPA, CT Pulmonary angiogram; nil acute, scans with no acute pulmonary pathology; PE, pulmonary embolus; other, patients with other pathologies on CTPA including lobar pneumonia, pleural effusion, etc.

The data also revealed differences in alternative lung parenchymal pathology identified on CTPA (Table 2). In 2019, 34.1% (*n* = 29/85) patients had no acute pulmonary pathology reported compared with 52.0% (*n* = 76/146) patients in 2020. Conversely, in 2019 49.4% (*n* = 42/85) patients were found to have an alternative cause for their symptoms from the CTPA scan compared with just 15.0% (*n* = 22/146) in 2020 (*P* = 0.010) (Table 2), possibly indicating milder or later stage COVID‐19 infection, with resolution of parenchymal changes.

CTPAs which were positive for PE in outpatients presenting in each time period were also reviewed to determine whether these patients had associated areas of parenchymal change. Overall, only 3.9% (*n* = 6/152) outpatient scans from 2020 and 4.5% (*n* = 4/89) scans from 2019 had a PE‐positive CTPAs (Table 2). In 2020, 5/6 patients with PE‐positive CTPAs had parenchymal changes, compared with 3/4 patients in 2019 (Table 2).

### Serological evaluation for COVID‐19 antibodies

Finally, correlation between PE‐negative CTPA findings in the 2020 cohort (*n* = 146) and COVID‐19 serology was evaluated. As COVID‐19 was not suspected as the primary diagnosis in this patient cohort, COVID‐19 PCR testing was not performed when they initially presented to hospital. In total, 39 patients (26.5%) of the PE‐negative cohort from 2020 attended for a COVID‐19 antibody test with a mean time of 101 days (standard deviation ± 30 days) between the CTPA and COVID‐19 antibody test. Only 3 patients tested positive for COVID‐19 antibodies with 2 of the antibody positive patients having areas of parenchymal change on CT. Of the 36 patients negative for COVID‐19 antibodies, 6 had likely COVID‐19 based on the CTPA BSTI grading, 6 had areas of parenchymal change, 19 had normal CTPAs, and 5 had an alternative cause for their symptoms. Notably, none of the 6 patients with radiologically likely COVID‐19 pneumonia who attended for antibody testing were positive for COVID‐19 antibodies (*n* = 0/6).

## Discussion

This study showed increased hospital presentation of suspected PE in 2020 compared to 2019, and in 2020, 32.8% of these patients had pulmonary parenchymal changes either characteristic of COVID‐19 or suggestive of viral infection, including 11 patients whose CTPA was likely/suspicious for COVID‐19. Most patients did not have typical COVID‐19 pneumonia symptoms except for a dry cough in 21.2% of cases, which is also a PE feature, but all presented with chest pain more typical of PE. More severe COVID‐19 cases are typically associated with reduced oxygen saturation, fever, lymphopenia and substantially elevated D‐Dimer and CRP values, which was not observed in our cohort. Although only 25.6% patients contacted attended for voluntary COVID‐19 antibody testing, none of the 6 patients with a CTPA which was likely/suspicious for COVID‐19 had COVID‐19 antibodies. Thus overall, the significant increase in parenchymal changes in 2020 compared with 2019 is likely to be related to COVID‐19 infection that was contained and thus may not have generated systemic humoral immune responses.

Fewer patients presented to hospital in 2020 compared with 2019, including fewer outpatient CTPAs, possibly reflecting public reticence to attend hospital during the pandemic. Similarly, pressures on hospital trusts to avoid any hospital admission where possible, in efforts to reduce nosocomial transmission of the disease, may account for the reduction in inpatient CTPAs in 2020 compared with 2019. However, interestingly whilst 154 outpatients in 2020 presented with chest pain compared with 89 in 2019, the number of confirmed PEs was similar. This further supports that chest pain may indeed by a presentation for mild COVID‐19 infection masquerading as suspected PE, and it may be reasonable to consider treating all patients with presentations such as this is as suspected COVID‐19.

We were unable to collect COVID‐19 PCR data for our patients due to the unavailability of COVID‐19 PCR testing for all outpatients in the trust, as it was not trust or indeed NHS national policy at the time to swab outpatients for COVID‐19 during the first wave of the COVID‐19 pandemic. This was largely due to lack of testing kits and/or infrastructure to conduct mass testing in the outpatient setting. However, we suggest that viral containment by pulmonary intravascular immune‐thrombosis may also constrain local and blood borne dissemination thereby preventing the immune system coming into contact with and mounting a response to COVID‐19. A recent systematic review found that the sensitivity of COVID‐19 nasopharyngeal swabs could be as low as 71%, particularly in milder cases of disease [14] and is heavily dependent on swab technique [14] and the time at which the individual is swabbed during their illness [14, 15].

Of the patients that attended for COVID‐19 antibody testing, very few were positive. This result may simply reflect the face that the low numbers of patients who attended for antibody testing (26.4%, 39/148). However, we still observed that 0/6 patients who met BSTI criteria were positive for COVID‐19 antibodies. This may reflect the known association with negative antibody testing results and mild disease [16, 17, 18, 19]. Existing antibody tests have been validated using severe cases; hence, the sensitivity of existing antibody tests is likely to be lower in milder cases of disease [20, 21]. Emerging data also suggests that detectable antibody responses decline rapidly following infection, particularly in mild cases [16], as previously observed for other human coronaviruses [17, 22]; thus, the time lag between presentation with chest pain and phlebotomy (mean 100 days) may explain the lack of detectable antibodies in our cohort. Finally, current antibody tests measure IgM and IgG titres; however, IgA plays a pivotal role in immunity to respiratory pathogens, and T‐cell immunity may also be present in the absence of a detectable antibody titre; however, we were unable to test for these in our patients [21, 23, 24, 25, 26]. Given the potential limitations of COVID‐19 antigen and antibody testing in mild disease, the findings of this study are relevant to better define the epidemiology of COVID‐19 disease and potential tracing and shielding for risk groups.

Our study shows that imaging may be a more reliable marker for COVID‐19 infection than PCR or antibody testing. We chose CTPAs since these are readily available in our trust and have been shown superior sensitivity for COVID‐19 diagnosis than PCR testing, or CXRs, and have the advantage of excluding concomitant PEs which are common in COVID‐19 patients. CTPAs are, however, associated with a significant radiation dose and may not be suitable for all patients, for example patients who are pregnant or have renal impairment. One alternative imaging modality that has been used in COVID‐19 diagnosis is lung ultrasound (LUS), which is rapid, noninvasive and low cost [27, 28]. Studies have shown it may be useful, particularly in the diagnosis of early disease and may correlate with disease severity [29, 30, 31]. On the other hand, it has variable sensitivity/specificity depending on the setting in which it is used (e.g. the emergency department versus critical care) [32, 33, 34] and facilities and expertise in this imaging modality are less than they are for CTPA in most UK hospitals, which may prevent more widespread use [35, 36].

### Limitations

There are some limitations including the relatively small cohort size and single‐centre retrospective nature; thus, initial findings require further validation. Due to trust policy at the time, only patients who were being admitted to hospital underwent COVID‐19 nasopharyngeal PCR testing. Outpatients, irrespective of whether or not they were considered to have possible COVID‐19, were not offered this test, and so, we are unable to correlate our CTPA findings with COVID‐19 PCR data. We attempted to collect data on COVID‐19 antibodies; however, there was poor uptake (25.6%), possibly due to patient reluctance to attend hospitals for nonurgent tests during the middle of the pandemic. Irrespective of this, antibody testing has other limitations. It is also impossible to determine when antibody positive patients may have been infected, and for patients who did test positive in this cohort, COVID‐19 infection may have occurred after, and be unrelated to, the index episode of chest pain. Our trust only reported antibody positivity/negativity and not the titre, and it is possible that our antibody test was unable to detect low titre antibodies that may have been present in some of our patients. This is particularly relevant as the mean time from index episode of chest pain and antibody tests is 100 days. Future studies should seek to collect COVID‐19 PCR at presentation and a complete immunological profile including CD4+ and CD8+ T‐cell responses and antibody responses checked 6 weeks after index presentation. Finally, the changes seen on CTPA are nonspecific and could still have be caused by other viral or bacterial pathogens or noninfective processes such as heart failure or malignancy, although the distribution of the changes, their focal nature and the differences observed in their frequency between 2019 and 2020 suggest otherwise.

## Conclusion

In conclusion, this study has shown an increased hospital outpatient attendance of ambulatory patients in 2020 with symptoms suggestive of PE without clinical or laboratory features of COVID‐19 but where CTPA demonstrated parenchymal changes consistent with or possible for COVID‐19 infection. These differences could represent focal COVID‐19 infection with localized immune‐thrombosis and infarction resulting in chest pain imitating PE. Without the characteristic radiological features, this group may pose a diagnostic dilemma as 0/6 patients whose CTPA was “likely/ suspicious for COVID‐19” were antibody positive. This has potentially significant importance for clinical practice and for developing our understanding of mild COVID‐19 pneumonia, as well as the pathophysiology, transmission and spread of COVID‐19.

## Conflicts of interest

HMO and DMG are supported by the National Institute for Health Research (NIHR) Leeds Biomedical Research Centre. AS receives salary support from Leeds Cares (Leeds Teaching Hospitals Charitable Foundation) and Innovate UK. The views expressed are those of the authors and not necessarily those of the (UK) National Health Service (NHS), the NIHR or the (UK) Department of Health.

## Author contribution

**Stephanie Rose Harrison:** Conceptualization (equal); Data curation (lead); Formal analysis (lead); Funding acquisition (equal); Investigation (lead); Methodology (equal); Project administration (lead); Visualization (lead); Writing‐original draft (lead); Writing‐review & editing (lead). **Joel RL Klassen:** Conceptualization (supporting); Data curation (supporting); Formal analysis (supporting); Investigation (supporting); Visualization (supporting); Writing‐original draft (supporting); Writing‐review & editing (supporting). **Charlie Bridgewood:** Conceptualization (supporting); Formal analysis (supporting); Methodology (supporting); Visualization (supporting); Writing‐original draft (supporting); Writing‐review & editing (supporting). **Andrew Scarsbrook:** Conceptualization (equal); Data curation (equal); Formal analysis (equal); Methodology (equal); Supervision (equal); Visualization (equal); Writing‐original draft (supporting); Writing‐review & editing (supporting). **Helena Marzo‐Ortega:** Conceptualization (equal); Funding acquisition (supporting); Investigation (supporting); Methodology (equal); Supervision (supporting); Visualization (equal); Writing‐original draft (equal); Writing‐review & editing (equal). **Dennis McGonagle:** Conceptualization (lead); Formal analysis (equal); Investigation (lead); Methodology (lead); Resources (equal); Supervision (lead); Visualization (equal); Writing‐original draft (lead); Writing‐review & editing (lead).

## References

[1] John Hopkins . COVID‐19 Dashboard. [cited 2020 Sep 22]. Available from: https://coronavirus.jhu.edu/map.html

[2] Pascarella G , Strumia A , Piliego C , Bruno F , Del Buono R , Costa F , *et al*. COVID‐19 diagnosis and management: a comprehensive review. J Intern Med. 2020;288:192–206. 32348588 10.1111/joim.13091 PMC7267177

[3] Middeldorp S , Coppens M , van Haaps TF , Foppen M , Vlaar AP , Müller MCA , *et al*. Incidence of venous thromboembolism in hospitalized patients with COVID‐19. J Thromb Haemost. 2020;18:1995–2002. 32369666 10.1111/jth.14888 PMC7497052

[4] Cui S , Chen S , Li X , Liu S , Wang F . Prevalence of venous thromboembolism in patients with severe novel coronavirus pneumonia. J Thromb Haemost. 2020;18:1421–4. 32271988 10.1111/jth.14830 PMC7262324

[5] Lang M , Som A , Carey D , Reid N , Dexter P , Flores EJ , *et al*. Pulmonary vascular manifestations of COVID‐19 Pneumonia. Radiol Cardiothorac Imaging. 2020;2:1–26. 10.1148/ryct.2020200277 PMC7307217 34036264

[6] McGonagle D , O’Donnell JS , Sharif K , Emery P , Bridgewood C . Immune mechanisms of pulmonary intravascular coagulopathy in COVID‐19 pneumonia. Lancet Rheumatol. 2020;2:e437–e445. 32835247 10.1016/S2665-9913(20)30121-1 PMC7252093

[7] Ackermann M , Verleden SE , Kuehnel M , Haverich A , Welte T , Laenger F , *et al*. Pulmonary vascular endothelialitis, thrombosis, and angiogenesis in Covid‐19. N Engl J Med. 2020;383:120–8. 32437596 10.1056/NEJMoa2015432 PMC7412750

[8] Levi M , Thachill J , Iba T , Levy JH . Coagulation abnormalities and thrombosis in patients with COVID‐19. Lancet Haematol. 2020;7:e438–e440. 32407672 10.1016/S2352-3026(20)30145-9 PMC7213964

[9] Koo HJ , Lim S , Choe J , Choi SH , Sung H , Do KH . Radiographic and CT features of viral pneumonia. Radiographics. 2018;38:719–39. 29757717 10.1148/rg.2018170048

[10] Gandhi RT , Lynch JB , del Rio C . Mild or moderate Covid‐19. N Engl J Med 2020;383:1757–66. 32329974 10.1056/NEJMcp2009249

[11] Watson J , Whiting PF , Brush JE . Interpreting a covid‐19 test result. BMJ. 2020;369:1–7. 10.1136/bmj.m1808 32398230

[12] British Society of Thoracic Imaging . **UPDATED** COVID‐19 BSTI REPORTING TEMPLATES AND CODES [Internet]. [cited 2020 May 20]. Available from: https://www.bsti.org.uk/covid‐19‐resources/covid‐19‐bsti‐reporting‐templates/

[13] UK Government . Coronavirus (COVID‐19): antibody testing [Internet]. [cited 2020 May 20]. Available from: https://www.gov.uk/government/publications/coronavirus‐covid‐19‐antibody‐tests/coronavirus‐covid‐19‐antibody‐tests

[14] Mohammadi A , Esmaeilzadeh E , Li T , Bosch RJ , Li JZ . SARS‐CoV‐2 detection in different respiratory sites: A systematic review and meta‐analysis. EBioMedicine. 2020;59:102903. 32718896 10.1016/j.ebiom.2020.102903 PMC7380223

[15] Sethuraman N , Jeremiah SS , Ryo A . Interpreting diagnostic tests for SARS‐CoV‐2. J Am Med Assoc. 2020;323:2249–51. 10.1001/jama.2020.8259 32374370

[16] Ibarrondo FJ , Fulcher JA , Goodman‐Meza D , Elliott J , Hofmann C , Hausner MA , *et al*. Rapid decay of anti–SARS‐CoV‐2 antibodies in persons with mild COVID‐19. N Engl J Med. 2020;1:10–2. 10.1056/NEJMc2025179 PMC7397184 32706954

[17] Watson J , Richter A , Deeks J . Testing for SARS‐CoV‐2 antibodies. BMJ. 2020;370:m3325. 32900692 10.1136/bmj.m3325

[18] Wang Y , Zhang L , Sang L , Ye F , Ruan S , Zhong B , *et al*. Kinetics of viral load and antibody response in relation to COVID‐19 severity. J Clin Invest. 2020;130:5235–44. 32634129 10.1172/JCI138759 PMC7524490

[19] Wang F , Hou H , Ying L , Tang G , Wu S , Huang M , *et al*. The laboratory tests and host immunity of COVID‐19 patients with different severity of illness. JCI Insight. 2020;5:e137799. 10.1172/jci.insight.137799 PMC7259533 32324595

[20] Deeks JJ , Dinnes J , Takwoingi Y , Davenport C , Spijker R , Taylor‐Philips S , *et al*. Antibody tests for identification of current and past infection with SARS‐CoV‐2. Cochrane Database Syst Rev. 2020;2:1–306. 10.1002/14651858.CD013652 PMC7387103 32584464

[21] Le Bert N , Tan AT , Kunasegaran K , Tham CYL , Hafezi M , Chia A , *et al*. SARS‐CoV‐2‐specific T cell immunity in cases of COVID‐19 and SARS, and uninfected controls. Nature. 2020;584:457–62. 32668444 10.1038/s41586-020-2550-z

[22] European Centre for Disease Prevention and Control . Immune responses and immunity to SARS‐CoV‐2 [Internet]. [Cited 19 June 2020]. Available from: https://www.ecdc.europa.eu/en/covid‐19/latest‐evidence/immune‐responses

[23] Azkur AK , Akdis M , Azkur D , Sokolowska M , van de Veen W , Brüggen M‐C , *et al*. Immune response to SARS‐CoV‐2 and mechanisms of immunopathological changes in COVID‐19. Allergy. 2020;75:1564–81. 32396996 10.1111/all.14364 PMC7272948

[24] Amanat F , Stadlbauer D , Strohmeier S , Nguyen THO , Chromikova V , McMahon M , *et al*. A serological assay to detect SARS‐CoV‐2 seroconversion in humans. Nat Med. 2020;26:1033–6. 32398876 10.1038/s41591-020-0913-5 PMC8183627

[25] Alisa F , Jessica M , Fatima A , Florian K , Jennifer H‐H , Susan O‐P , *et al*. Evidence of a significant secretory‐IgA‐dominant SARS‐CoV‐2 immune response in human milk following recovery from COVID‐19. medRxiv 2020.

[26] Ma H , Zeng W , He H , Zhao D , Jiang D , Zhou P , *et al*. Serum IgA, IgM, and IgG responses in COVID‐19. Cell Mol Immunol. 2020;17:773–5. 32467617 10.1038/s41423-020-0474-z PMC7331804

[27] Piliego C , Strumia A , Stone MB , Pascarella G . The ultrasound‐guided triage: a new tool for prehospital management of COVID‐19 pandemic. Anesth Analg. 2020;131:e93–e94. 32345853 10.1213/ANE.0000000000004920 PMC7202119

[28] Pascarella G , Strumia A , Stone MB , Piliego C . The evolution of ultrasound role in COVID‐19 pandemic: from triage to screening. Anesth Analg. 2020 [ePub ahead of print]. 10.1213/ANE.0000000000005143 PMC7202119 32345853

[29] Lopes AJ , Mafort TT , da Costa CH , Rufino R , de Cássia FM , Kirk KMC *et al*. Comparison between lung ultrasound and computed tomographic findings in patients With COVID‐19 pneumonia. J Ultrasound Med. 2020 [ePub ahead of print]. 10.1002/jum.15521 PMC7537266 32996607

[30] Knight T , Edwards L , Rajasekaran A , Clare S , Lasserson D . Point‐of‐care lung ultrasound in the assessment of suspected COVID‐19: a retrospective service evaluation with a severity score. Acute Med. 2020;19:192–200. 33215172

[31] Nouvenne A , Zani MD , Milanese G , Parise A , Baciarello M , Bignami EG , *et al*. Lung ultrasound in COVID‐19 pneumonia: correlations with chest CT on Hospital admission. Respiration. 2020;99:617–24. 32570265 10.1159/000509223 PMC7360505

[32] Schmid B , Feuerstein D , Lang CN , Fink K , Steger R , Rieder M , *et al*. Lung ultrasound in the emergency department ‐ a valuable tool in the management of patients presenting with respiratory symptoms during the SARS‐CoV‐2 pandemic. BMC Emerg Med. 2020;20:96. 33287732 10.1186/s12873-020-00389-w PMC7720034

[33] Haak SL , Renken IJ , Jager LC , Lameijer H , van der Kolk BBY . Diagnostic accuracy of point‐of‐care lung ultrasound in COVID‐19. Emerg Med J. 2020;38:94–9. 33208399 10.1136/emermed-2020-210125

[34] Narinx N , Smismans A , Symons R , Frans J , Demeyere A , Gillis M . Feasibility of using point‐of‐care lung ultrasound for early triage of COVID‐19 patients in the emergency room. Emerg Radiol. 2020;27:663–70. 32910323 10.1007/s10140-020-01849-3 PMC7481756

[35] Wolfshohl J , Shedd A , Chou EH , d'Etienne JP . Lung ultrasound for COVID‐19 evaluation in the emergency department: is it feasible? Ann Emerg Med. 2020;76:552–3. 33012385 10.1016/j.annemergmed.2020.05.033 PMC7254000

[36] Jackson K , Butler R , Aujayeb A . Lung ultrasound in the COVID‐19 pandemic. Postgraduate Med J. 2021;97:34–9. 10.1136/postgradmedj-2020-138137 PMC10016966 32895294

